# Fbxw11 impairs the repopulation capacity of hematopoietic stem/progenitor cells

**DOI:** 10.1186/s13287-022-02926-9

**Published:** 2022-06-11

**Authors:** Lina Wang, Yongjun Piao, Dongyue Zhang, Wenli Feng, Chenchen Wang, Xiaoxi Cui, Qian Ren, Xiaofan Zhu, Guoguang Zheng

**Affiliations:** 1 State Key Laboratory of Experimental Hematology, National Clinical Research Center for Blood Diseases, Haihe Laboratory of Cell Ecosystem, Institute of Hematology & Blood Disease Hospital, Chinese Academy of Medical Sciences & Peking Union Medical College, 288 Nanjing Road, Tianjin, 300020 China; 2 School of Medicine, Nankai University, Tianjin, China

**Keywords:** Fbxw11, Hematopoietic stem cell, Hematopoietic progenitor cell, Single-cell RNA sequencing

## Abstract

**Background:**

The ubiquitin–proteasome system plays important roles in maintaining the self-renewal and differentiation of stem and progenitor cells through highly ordered degradation of cellular proteins. Fbxw11, an E3 ligase, participates in many important biological processes by targeting a broad range of proteins. However, its roles in hematopoietic stem/progenitor cells (HSPCs) have not been established.

**Methods:**

In this study, the effects of Fbxw11 on HSPCs were studied in vitro and in vivo by an overexpression strategy. Real-time PCR was performed to detect the expression of Fbxw11 in hematopoietic subpopulations. Colony-forming assays were performed to evaluate the in vitro function of Fbxw11 on HSPCs. Hoechst 33342 and Ki67 staining was performed to determine the cell-cycle distribution of HSPCs. Competitive transplantation experiments were used to evaluate the effect of Fbxw11 on the reconstitution potential of HSPCs. Single-cell RNA sequencing (scRNA-seq) was employed to reveal the transcriptomic alterations in HSPCs.

**Results:**

The expression of Fbxw11 was higher in Lin^−^c-Kit^+^Sca-1^+^ (LSK) cells and myeloid progenitors than in lymphoid progenitors. Fbxw11 played negative roles in colony-forming and quiescence maintenance of HSPCs in vitro. Furthermore, serial competitive transplantation experiments revealed that Fbxw11 impaired the repopulation capacity of HSPCs. The proportion of granulocytes (Gr-1^+^CD11b^+^) in the differentiated mature cells was significantly higher than that in the control group, T cells and B cells were lower. Moreover, scRNA-seq revealed seven cell clusters in HSPCs. In addition, Fbxw11 downregulated the expression of *Cebpa*, *Myc* and *Arid5b*, which are significant regulators of HSPC activity, in most cell clusters.

**Conclusion:**

Our data demonstrate that Fbxw11 plays a negative role in the maintenance of HSPCs in vitro and repopulation capacity in vivo*.* Our data also provide valuable transcriptome references for HSPCs in homeostasis.

**Supplementary Information:**

The online version contains supplementary material available at 10.1186/s13287-022-02926-9.

## Introduction

Self-renewal and differentiation are two essential functions of hematopoietic stem cells (HSCs) to maintain the homeostasis of the entire hematopoietic system under physiologic and pathologic conditions [1]. These two functions are well balanced by sophisticated mechanisms [2, 3], and breaking the balance results in blood diseases [4, 5]. The ubiquitin–proteasome system (UPS), which controls the degradation of proteins with a short half-time, is a key player in maintaining the balance and is involved in normal and malignant hematopoiesis [6]. F-box family proteins, which are further divided into Fbxw, Fbxl and Fbxo subfamilies based on protein structure, determine the specificity of UPS degradation by identifying a specific spectrum of target proteins for ubiquitination [7]. F-box family proteins are widely involved in hematopoietic regulation, and their abnormal expression or dysfunction results in deteriorations in hematopoietic homeostasis and even malignancies [8]. Fbxw7 maintains HSC quiescence to preserve self-renewal potential in normal hematopoiesis [9]. Fbxw7, Fbxl2, Fbxl10 and SKP2 regulate the proliferation of leukemia cells through the ubiquitination pathway [10–12]. Loss-of-function mutations of *Fbxw7* are involved in both leukemogenesis and leukemic progression by affecting the ubiquitination and half-life of c-Myc protein, which is essential for the maintenance of HSC quiescence [13, 14]. Deletions or loss-of-function mutations of *Fbxo11* contribute to the pathogenesis of diffuse large B-cell lymphomas [15]. However, the roles of F-box proteins in hematopoiesis are not fully understood.

Fbxw11 participates in many important biological processes, including proliferation, differentiation, development and metabolism, by targeting a broad range of substrates for degradation [16, 17]. Fbxw11 is involved in cell proliferation by modulating the activities of cycle-related transcription factors and regulators. However, the effects of Fbxw11 on cell proliferation, either stimulatory or inhibitory, seem to be cell type dependent since the substrates of Fbxw11 cover both cell cycle activator and suppressors. In our previous studies, Fbxw11 is upregulated in acute lymphocytic leukemia, and high level of Fbxw11 expression promotes the proliferation of leukemia cells [18]. Cell cycle regulation is very important for HSCs to maintain self-renewal and engraftment potential. Hence, how Fbxw11 impacts the proliferation, repopulation and self-maintenance of normal HSPCs is of interest. Furthermore, HSPCs are heterogeneous and can be divided into functionally hierarchical subpopulations [19]. Recent evidence defined HSPC subpopulations by their transcriptomic landscapes and revealed the diversity among them at single-cell resolution [20–23]. However, how Fbxw11 affects HSPC subpopulations at single-cell resolution has not been elucidated.

In the present study, the in vitro and in vivo effects of Fbxw11 on HSPCs were studied by overexpressing Fbxw11 in HSPCs. Furthermore, single-cell RNA sequencing (scRNA-seq) was employed to explore the transcriptomic heterogeneity of HSPCs and gene expression profiling alterations by Fbxw11 at the single-cell level. This work contributes to the comprehensive understanding of the effects of Fbxw11 on hematopoietic regulation and provides valuable transcriptome references for HSPCs in homeostasis.

## Materials and methods

### Mice

Seven- to eight-week-old female B6.SJL (CD45.1) and C57BL/6 (CD45.2) congenic mice were provided by the Animal Centre of the Institute of Hematology and Blood Diseases Hospital, CAMS & PUMC, and maintained in an SPF-certified facility. All procedures for animal experiments were approved by the Institutional Animal Care and Use Committee at the institute.

### Vectors

The retroviral vector pMSCV-PGK-GFP was previously used in our laboratory [24, 25]. The recombinant vectors were constructed by inserting murine Fbxw11 variants into pMSCV-PGK-GFP. The blank vector was used as a control.

### Isolation of cells

Bone marrow (BM) cells were obtained from the tibia and femur of 8-week-old mice. The markers and strategies for the isolation of hematopoietic cells were described previously [25–29]. For Lin^−^c-Kit^+^Sca-1^+^ (LSK) cells, Lin^−^c-kit^+^ (LK) cells, long-term hematopoietic stem cells (LT-HSCs), short-term hematopoietic stem cells (ST-HSCs), multipotent progenitors (MPPs), common myeloid progenitors (CMPs), granulocyte–monocyte progenitors (GMPs), megakaryocyte–erythroid progenitors (MEPs) and common lymphoid progenitors (CLPs), the c-kit^+^ cells were first enriched with microbeads according to the manufacturer’s guidelines (Miltenyi Biotec, Bergisch Gladbach, Germany, 130–091-224). Then, cells were stained with V450-Lin (Ter119, Gr-1, CD11b, B220, CD3, CD4, CD8), PE-Cy7-c-Kit, APC-Sca-1, Percp-Cy5.5-CD34, PE-Flk2, PE-CD16/32 and PE-IL7R antibodies before flow cytometric cell sorting following the strategies shown in Additional file 1: Fig. S1A–B. For granulocytes, macrophages and monocytes, BM cells were stained with Percp-Cy5.5-CD3, Percp-Cy5.5-CD19, BV-421-Gr-1, APC-Cy7-CD11b, PE-CD115 and APC-F4/80 antibodies before flow cytometric cell sorting following the strategies shown in Additional file 1: Fig. S1C–D. For B cells, T cells and NK cells, splenic cells were stained with Percp-Cy5.5-CD3, PE-B220, PE-Cy7-CD4, APC-Cy7-CD8, PE-Cy7-NK1.1 and APC-NKP46 before flow cytometric cell sorting following the strategies shown in Additional file 1: Fig. S1E–F. All antibodies are summarized in Additional file 2: Table S1, and cell staining was performed following standard protocols.

### Retroviral infection

The retroviruses were harvested following standard protocols. Prior to virus infection, LSK or LK cells from C57BL/6 mice were prestimulated for 16 h in StemSpan SFEM (STEMCELL Technologies, Vancouver, BC, Canada) supplemented with 100 U/ml penicillin, 100 mg/ml streptomycin, 100 ng/ml SCF, 20 ng/ml TPO, 10 ng/ml IL-3 and 10 ng/ml IL-6. (All cytokines were purchased from PeproTech, Rocky Hill, NJ.) Then, the cells were infected twice in 2 ml of virus stock containing 8 µg/ml polybrene at one-day intervals. After sorting by flow cytometry, GFP^+^ LSK cells were named LSK-Fbxw11c, LSK-Fbxw11d and LSK-Con.

### Flow cytometric analysis and cell sorting

Cells were resuspended for FACS analysis using a FACS Canto II or sorting using a FACS ARIA III (BD Biosciences, San Jose, CA) following standard protocols. The FACS data were analyzed using FlowJo software or BD FACS Diva Software.

### Colony-forming assay

GFP^+^ cells were sorted 48 h after LSK cells were infected with recombinant or control retrovirus. Five hundred GFP^+^ cells were plated in each well in MethoCult GF M3434 medium (Stem Cell Technologies, Vancouver, BC, Canada) and cultured for 10 days according to the manufacturer’s instructions. Colonies were counted under a microscope.

### Homing assay

GFP^+^ cells were sorted 48 h after LK cells were infected with recombinant or control retrovirus. GFP^+^ cells (1 × 10^6^) were transplanted into lethally irradiated (9.5 Gy) recipient mice (CD45.2). After 16 h, the recipient mice were killed, and the BM GFP^+^ donor cells were analyzed by FACS.

### Competitive transplantation assay

GFP^+^ LSK cells were sorted 48 h after LSK were infected with recombinant or control retrovirus. In the first round of transplantation, 1 × 10^4^ GFP^+^ LSK cells (CD45.1^+^) were transplanted with 2 × 10^5^ competitor cells (CD45.2^+^ BM cells) into lethally irradiated recipients (CD45.2). The percentage of GFP^+^ cells in the peripheral blood (PB) was monitored monthly by FACS for 5 months. The mice were killed, and BM cells were collected. The percentage of different lineages in GFP^+^ cells was assessed by FACS. In the second round of transplantation, 2 × 10^5^ GFP^+^ cells from the first round of recipients (CD45.1^+^) were transplanted with 2 × 10^5^ competitor cells (CD45.2^+^) into lethally irradiated female recipients (CD45.2^+^).

### Cell cycle analysis

The cell cycle of GFP^+^ LSK cells was analyzed 48 h after LSK cells were infected with recombinant or control retrovirus. Briefly, cells were first stained with V450-Lineage, PE-Cy7-c-kit and APC-Sca-1. Then, cells were treated with Cytofix/Cytoperm buffer followed by Perm/Wash buffer (BD Pharmingen) and stained with PE-Ki-67. Finally, cells were resuspended in staining buffer with Hoechst 33342 (Sigma) before analysis by FACS following standard protocols.

### Real-time RT-PCR

Total RNA was extracted from GFP^+^ LSK cells that had been infected with recombinant or control retrovirus using TRIzol Reagent (ThermoFisher, Carlsbad, CA, USA). cDNA was synthesized using M-MLV reverse transcriptase (Thermo Fisher) according to the manufacturer’s instructions. Real-time PCR was performed using an ABI Q5 Sequence Detector System (Applied Biosystems, Foster City, CA). The relative level of target genes was calculated using the ^ΔΔ^Ct method. The sequences of all primers are listed in Additional file 3: Table S2.

### Western Blot

Total protein was extracted from LSK-Fbxw11c, LSK-Fbxw11d and LSK-Con using lysis buffer (Cell Signaling Technology, Danvers, MA) supplemented with protease inhibitors 72 h after retroviral infection. Proteins were separated by 10% SDS–polyacrylamide gel electrophoresis and transfer to polyvinylidene difluoride (PVDF) membrane. PVDF membranes were incubated with diluted primary antibodies (1:1000 for Fbxw11; ab188511, Abcam, Cambridge, UK) and 1:5000 for glyceraldehyde 3-phosphate dehydrogenase (GAPDH; 97166, Cell Signaling, Danvers, MA) in TBST buffer containing 5% milk at 4℃ overnight. ChemiDoc^™^ imaging system (Bio-Rad) was used for chemiluminescent visualization of proteins after incubating with secondary antibodies. Results were quantified by gray value analysis (Image J).

### 10× Genomics single-cell RNA sequencing (scRNA-seq)

GFP^+^ LSK cells were collected 48 h after LSK were infected with recombinant or control retrovirus. Cell viability was assessed by an TC10 automated cell counter (Bio-Rad). Then, the library preparation procedures were performed according to the instructions of the manufacturer of the Chromium Single Cell 3′ Reagent Kit v2. (10X Genomics, Inc). The libraries were quantified and sequenced via Illumina NextSeq 550 at Novogene Co., Ltd., Beijing, China. The RNA-seq data that support the findings of this study have been deposited in the Gene Expression Omnibus (GEO) under accession number GSE160643.

### scRNA-seq-based data analysis

The quality of paired-end raw reads was checked with FastQC, and the low-quality reads, adapter sequences and reads that could not form pairs were removed using Trimmomatic [30]. Cell Ranger software (version 3.1.0, 10X Genomics) was then used to process alignment, barcode and UMI counting. The cleaned reads were mapped to the UCSC mouse reference genome (mm10) using the STAR [31] program that was included in the Cell Ranger pipeline, and the reads were separated into exonic (overlap with an exon > 50%), intronic (non-exonic and overlap with an intron) and intergenic reads based on the transcript annotation GTF (GRCm38 Ensembl 84). The exonic locus was prioritized when the reads were aligned to multiple loci. The exonic reads were further aligned to the annotated transcripts, and the reads that were uniquely mapped to the transcriptome were used for UMI counting to generate a gene-barcode matrix. Downstream data analysis was performed with the filtered gene-barcode matrix using the Seurat (version 3.0) R package [32]. Low-quality cells or empty droplets that had fewer than 500 genes and genes that were expressed in fewer than 3 cells were excluded, and 2000 highly variable genes identified using local polynomial regression were selected for the next step. Next, a linear transformation was used to scale the expression of each gene, and principal component analysis was performed on scaled data for dimension reduction. The number of statistically significant principal components was determined by ElbowPlot. A K-nearest neighbor graph was constructed based on Euclidean distance in reduced dimension space, and the Louvain algorithm with the resolution parameter 0.4 was applied to identify different clusters. The *t*-distributed stochastic neighbor embedding (t-SNE) was used to visualize the clusters. Differential expression analysis was performed using a Wilcoxon rank sum test to identify cluster-specific markers, and the differentially expressed genes (DEGs) were reported if the adjusted *p* value was < 0.05 and the log fold change was greater than 0.25. The scaled expression levels of markers across different clusters are displayed in a heatmap.

Monocle2 [33] was used to perform single-cell trajectory analysis. The Seurat object was converted into Monocle2 object followed by the calculation of normalization factor and dispersion. Then, DEGs were calculated and we selected DEGs with *q*-value < 0.01 for dimension reduction. Finally, we ordered the cells and got the pseudotime trajectory.

### Statistical analysis

All quantitative data are presented as the means ± SD. A *t* test was used to compare two sets of quantitative data. One-way analysis of variance (ANOVA) followed by Dunnett’s post hoc multiple comparison test was used for univariate comparisons among multiple sets. Two-way ANOVA followed by Bonferroni’s post hoc test was also used to analyze multiple factors. Data were analyzed using GraphPad Prism software. Two-sided *p* values < 0.05 were considered statistically significant.

## Results

### Distribution of Fbxw11 in hematopoietic cell populations

**Fig. 1:**
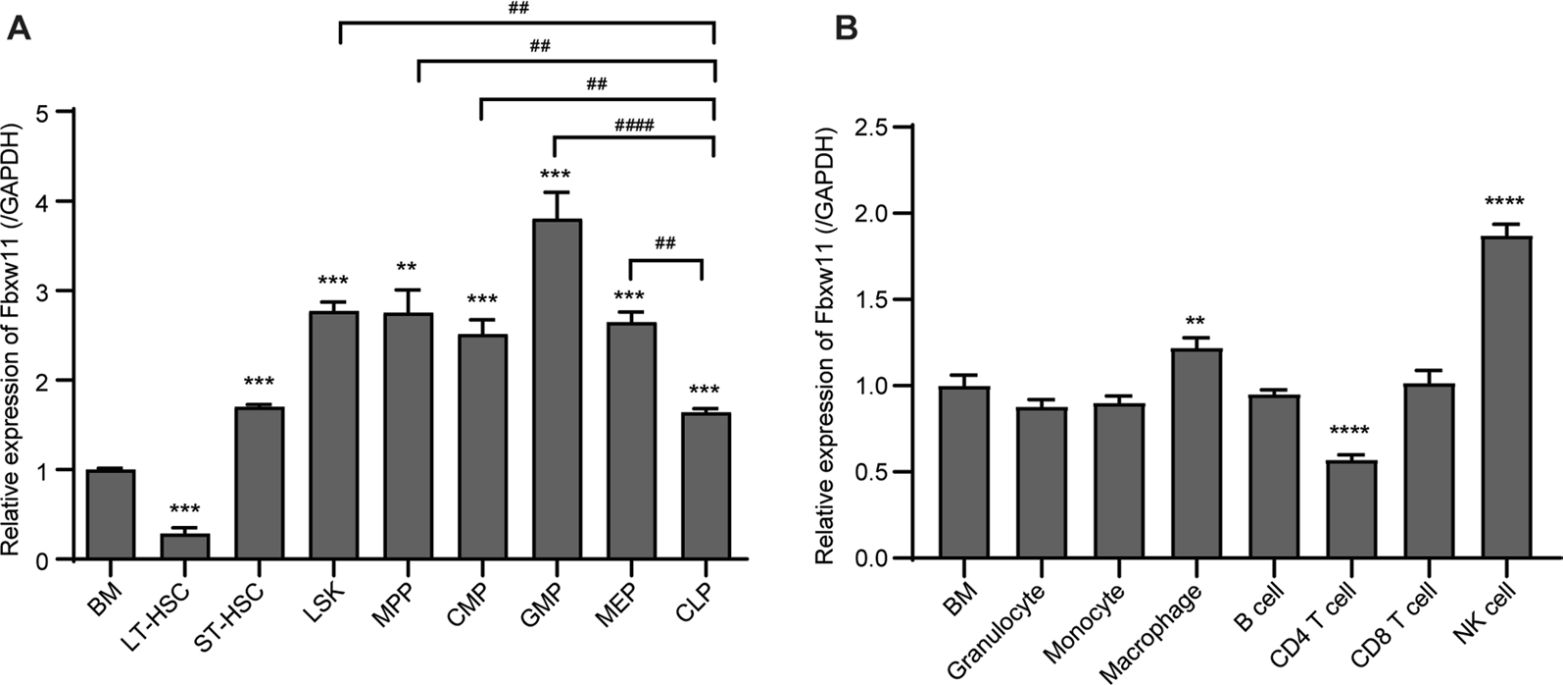
The expression of Fbxw11 in hematopoietic cells. **A** The relative expression of Fbxw11 to GAPDH in sorted LT-HSCs and ST-HSCs. LSK, MPPs, CMPs, GMPs, MEPs, CLPs and whole BM cells of C57BL/6 mice were studied by quantitative real-time PCR. **B** The expression of Fbxw11 in mature blood cells was studied by quantitative real-time PCR. The results were obtained from three independent experiments. *p < 0.05, **p < 0.01, ***p < 0.001

The hematopoietic cell populations were sorted by FACS following the strategies shown in Additional file 1: Fig. S1. The expression of Fbxw11 (covering all isoforms) in HSPC subpopulations, including LT-HSCs, ST-HSCs, LSK cells, MPPs, CMPs, GMPs, MEPs and CLPs, was first studied by quantitative real-time PCR. The expression of Fbxw11 was lower in purified murine LT-HSCs than in total BM samples. However, higher levels of Fbxw11 expression were observed in purified ST-HSCs, LSK cells, MPPs, CMPs, GMPs and MEPs, especially in GMPs, than in BM samples. Among the committed progenitors, Fbxw11 was expressed at higher levels in MPPs, GMPs and MEPs than in CLPs (Fig. 1A). Then, the expression of Fbxw11 in mature blood cells was examined. The results showed that higher levels were observed in macrophages and NK cells, whereas lower levels were detected in CD4 T cells (Fig. 1B). Collectively, the results show that Fbxw11 is differentially expressed among HSPC subpopulations, which suggests that Fbxw11 may play different roles among HSPC subpopulations.

### Fbxw11 plays a negative role in the maintenance of HSPCs in vitro

**Fig. 2:**
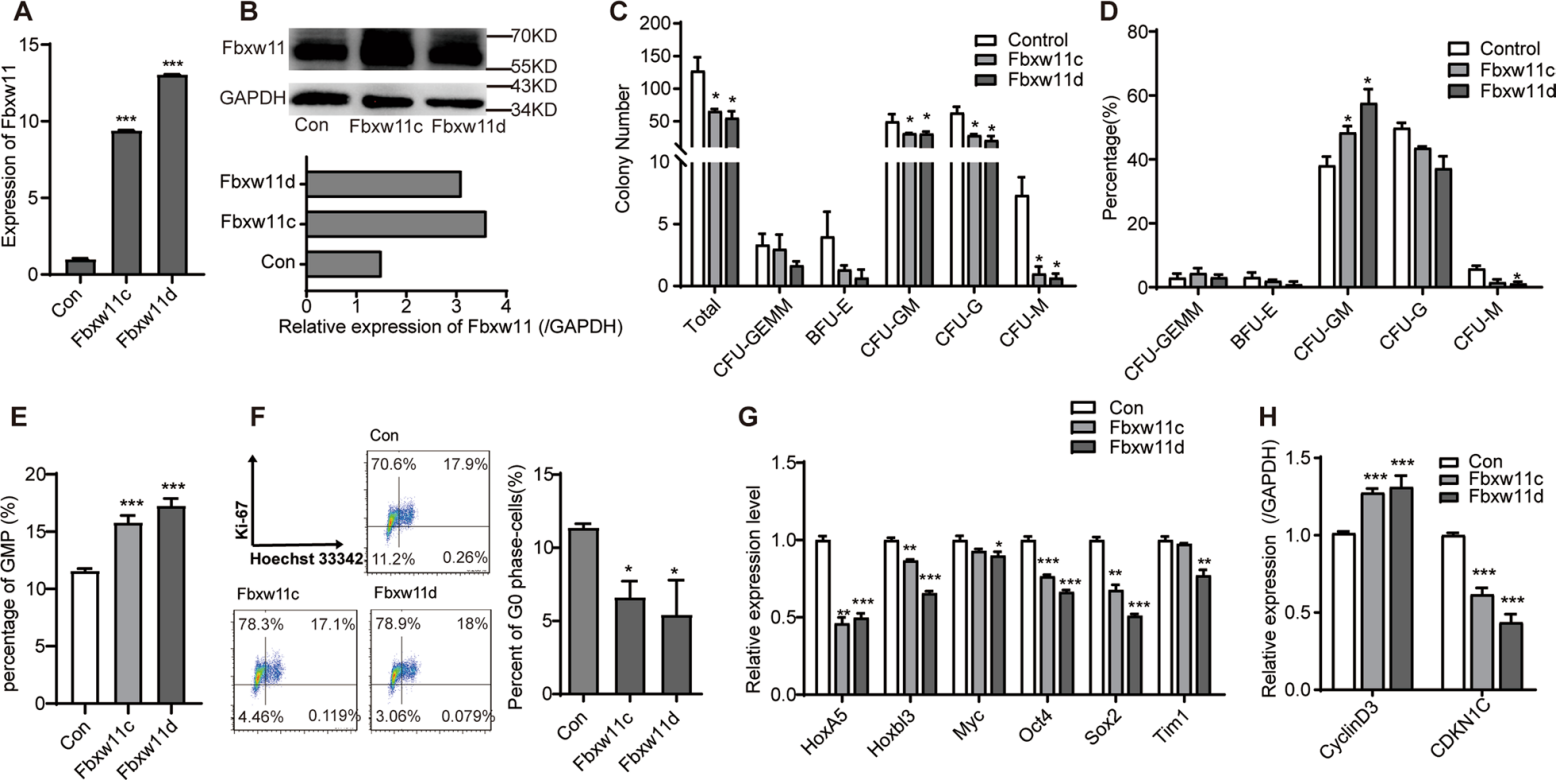
Fbxw11 plays a negative role in the maintenance of HSPCs in vitro. **A**, **B** LSK cells expressing high levels of Fbxw11 variants were established by retroviral infection and verified by real-time RT-PCR (**A**) and Western Blotting (**B**). **C**, **D** Colony-forming capacity of LSK cells overexpressing Fbxw11 variants was analyzed by colony-forming assays. The numbers of total colonies as well as GEMM, BFU-E, GM, G and M colonies (**C**) and the percentages (**D**) are plotted, *n* = 3. **E** Percentage of GMPs was tested by FACS 48 h after LSK cells were infected with retroviruses. **F** LSK cells overexpressing Fbxw11 variants were stained with Ki-67 and Hoechst 33342. Typical FACS results and the columns of the percentage of G0 phase LSK cells (bottom left quadrant) are shown, *n* = 3. **G**–**H** The expression of stem cell homeostasis-associated genes (**G**) and proliferation-associated genes (**H**) in LSK cells overexpressing Fbxw11 variants was studied by quantitative real-time PCR, *n* = 3. Data are presented as mean ± SEM, three independent experiments. *p < 0.05, **p < 0.01, ***p < 0.001

Although Fbxw11 is expressed as three transcript variants in humans and four variants in mice through alternative splicing [34, 35], only *Fbxw11c* and *Fbxw11d* were expressed mouse BM samples [18]. Therefore, LSK cells were transfected with recombinant retroviruses carrying Fbxw11c and Fbxw11d, respectively. High-level expression of Fbxw11 in LSK-Fbxw11c and LSK-Fbxw11d was verified by real-time RT-PCR and Western Blot (Fig. 2A, B). Colony-forming assays were performed to evaluate the in vitro function of LSK-Fbxw11c and LSK-Fbxw11d cells. Fbxw11 variants decreased the number of total colonies as well as GM, G and M colonies, indicating that Fbxw11 impaired the colony-forming ability of LSKs in vitro (Fig. 2C). However, the inhibition degrees varied among different kinds of colonies. An increase in the proportion of CFU-GM colonies in total colonies was observed (Fig. 2D). Analysis of GMPs 48 h after LSK cells were infected with retroviruses also demonstrated that Fbxw11 variants increased the percentage of GMPs (Fig. 2E). In order to know whether the in vitro impaired the maintenance of HSPCs by Fbxw11 variants was associated with changes in cell quiescence, a fundamental characteristic of stem cells, Ki-67 and Hoechst 33342 staining experiments were performed. The results demonstrated that the percentage of G0 stage cells (Ki-67^−^) was lower in LSK-Fbxw11c or LSK-Fbxw11d than in LSK-Con, suggesting that Fbxw11 promoted HSPCs to exit the quiescent state (Fig. 2F). Furthermore, the expression of stem cell homeostasis and proliferation-associated genes was evaluated. Interestingly, the expression levels of *HoxA5, Hoxbl3, Myc, Oct4, Sox2* and *Tim1* were significantly decreased in LSK-Fbxw11c and LSK-Fbxw11d (Fig. 2G). By contrast, upregulation of *CyclinD3*, which plays positive roles in proliferation, and downregulation of *Cdkn1c*, which plays negative roles in proliferation, were detected in LSK-Fbxw11c and LSK-Fbxw11d (Fig. 2H). These studies suggest that Fbxw11 may play a negative role in the maintenance of HSPCs in vitro, which may be associated with decreased quiescence of HSPCs.

### Fbxw11 reduces the in vivo repopulating capacity of HSPCs at steady state

**Fig. 3:**
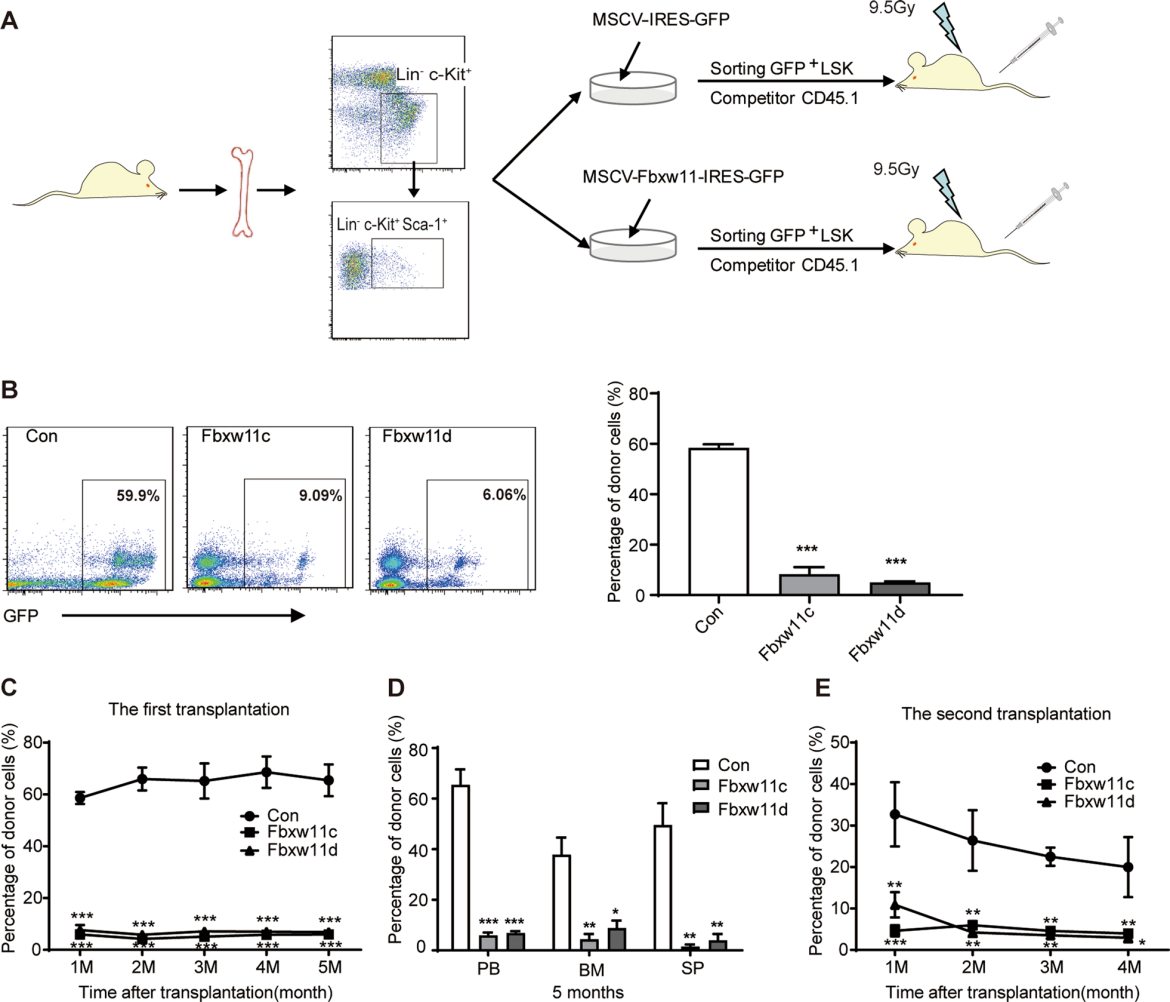
Fbxw11 reduces the in vivo repopulating capacity of HSPCs at steady state. **A** Experimental design of competitive reconstitution experiments. **B** The PB engraftment of GFP^+^ donor cells in recipient mice was detected by FACS one month after transplantation. The typical FACS results are shown (on the left), and the reconstitution efficiency is plotted (on the right), *n* = 5. **C** The PB engraftment of GFP^+^ donor cells in recipient mice was monitored monthly by FACS for 5 months, *n* = 5. **D** Mice were killed five months after first-round transplantation, and the engraftment of GFP^+^ cells in PB, BM and spleen cells was analyzed by FACS, *n* = 5. **E** GFP^+^ cells were sorted from the BM of first-round recipient mice five months after transplantation and transplanted into second-round recipient mice with competitor cells. The PB engraftment of GFP^+^ cells was monitored monthly for 4 months by FACS, *n* = 5. The data are representative of three independent experiments. *p < 0.05, **p < 0.01, ***p < 0.001

Competitive transplantation experiments were used to evaluate the effect of Fbxw11 on the reconstitution potential of HSPCs. The experimental design is shown in Fig. 3A. LK cells were used for the homing assay. The results showed that Fbxw11 variants had little effect on the homing capacity of LK cells (Additional file 1: Fig. S2A). LSK cells were used for a competitive transplantation assay. Fbxw11 variants decreased the repopulation potential of LSK cells one month after transplantation as assessed by the percentage of PB GFP^+^ donor cells in recipient mice (Fig. 3B). The reconstruction potential of LSK cells was further monitored by PB GFP^+^ cells for five months, showing that Fbxw11 variants decreased the engraftment of LSK cells throughout the experimental period (Fig. 3C). The reconstitution levels in the BM and spleen were assessed as GFP^+^ cells five months after transplantation. Lower reconstitution levels were observed in the Fbxw11 groups than in the control group (Fig. 3D). The overall reconstitution status (GFP^+^ cells and competitor cells) was assessed at the 1st, 3rd and 5th month after first-round transplantation, and no significant differences were detected among the three groups (Additional file 1: Fig. S2B–D). GFP^+^ cells from first-round recipients were isolated five months after the first BM transplantation and transplanted into secondary recipients. PB engraftment was monitored for 4 months. Fbxw11 variants significantly decreased long-term reconstitution in the secondary recipients (Fig. 3E). The reconstitution of BM donor-origin LSK cells was analyzed to see the effects of Fbxw11 on HSPCs. As expected, the reconstitution rate of LSK cells in Fbxw11-overexpressing group was significantly lower than the control group (Additional file 1: Fig. S2E). Taken together, these observations suggest that Fbxw11 reduces the repopulating capacity of HSPCs and long-term self-renewal of HSCs in vivo.

### Effects of Fbxw11 on the proportion of differentiated mature cells after transplantation

**Fig. 4:**
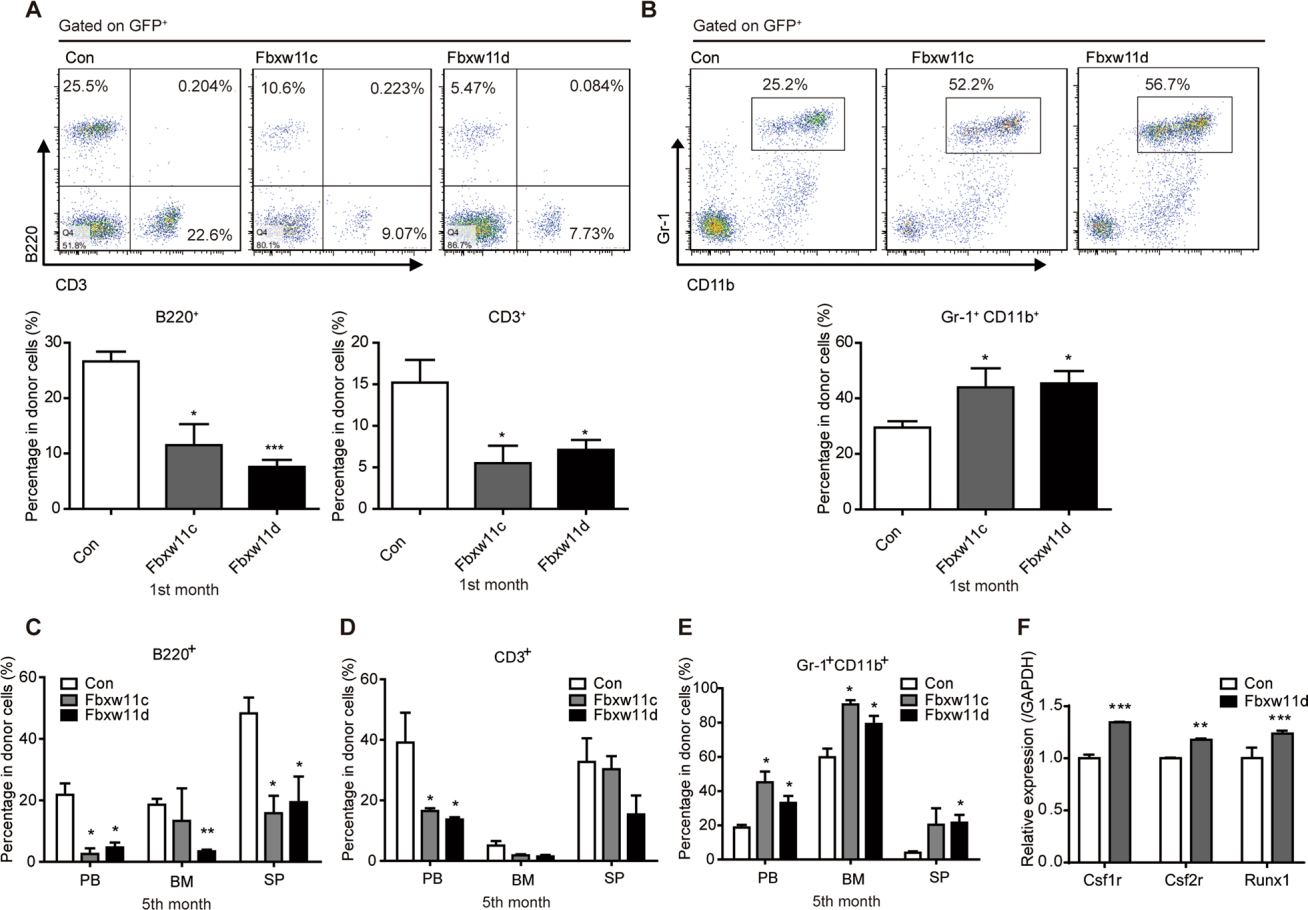
Lineage differentiation of HSPCs by Fbxw11 overexpression. **A**, **B** The lineages of donor-derived PB GFP^+^ cells were analyzed by FACS one month after first-round transplantation. The percentages of B220^+^ B cells, CD3^+^ T cells (**A**) and Gr-1^+^CD11b^+^ granulocytes (**B**) are shown. Typical FACS results (upper) and the percentages (lower) are shown. **C**–**E** The lineages of donor-derived PB GFP^+^ cells were analyzed five months after first-round transplantation. The percentages of B220^+^ B cells (**C**), PB CD3^+^ T cells (**D**) and Gr-1^+^CD11b^+^ granulocytes (**E**) are shown. **F** LSK cells were infected with control or recombinant retrovirus. LSK cells were sorted after 48 h, and the expression of myeloid-associated genes was studied by quantitative real-time PCR. *p < 0.05, **p < 0.01, ***p < 0.001

Multilineage differentiation is a key biological property of HSCs. The PB GFP^+^ donor cells in the competitive transplantation experiments were further analyzed by lineages one month after first-round transplantation. The percentage of B220^+^ B lymphocytes and CD3^+^ T lymphocytes was lower in the LSK-Fbxw11c or LSK-Fbxw11d group than in the control group (Fig. 4A), whereas the percentage of Gr-1^+^ CD11b^+^ granulocytes was higher in the LSK-Fbxw11c or LSK-Fbxw11d group than in the control group (Fig. 4B). GFP^+^ donor cells from the PB, BM and spleen were also assessed by lineages five months after first-round transplantation. Similar results were obtained (Fig. 4C–E). It was reported that CSF1R, CSF2R and RUNX1 are important regulators of myeloid cells. Upregulation of *Csf1r*, *Csf2r* and *Runx1* was detected in the LSK-Fbxw11c and LSK-Fbxw11d groups (Fig. 4F). Collectively, these data demonstrated that the effects of Fbxw11 on myeloid and lymphoid cells varied and implied that Fbxw11 might play more important roles in myeloid cells than lymphoid cells. Fbxw11d was chosen for further study hereafter since the above results demonstrated little difference between Fbxw11c and Fbxw11d.

### Single-cell analysis of the effects of Fbxw11 on heterogeneous HSPC subpopulations

**Fig. 5:**
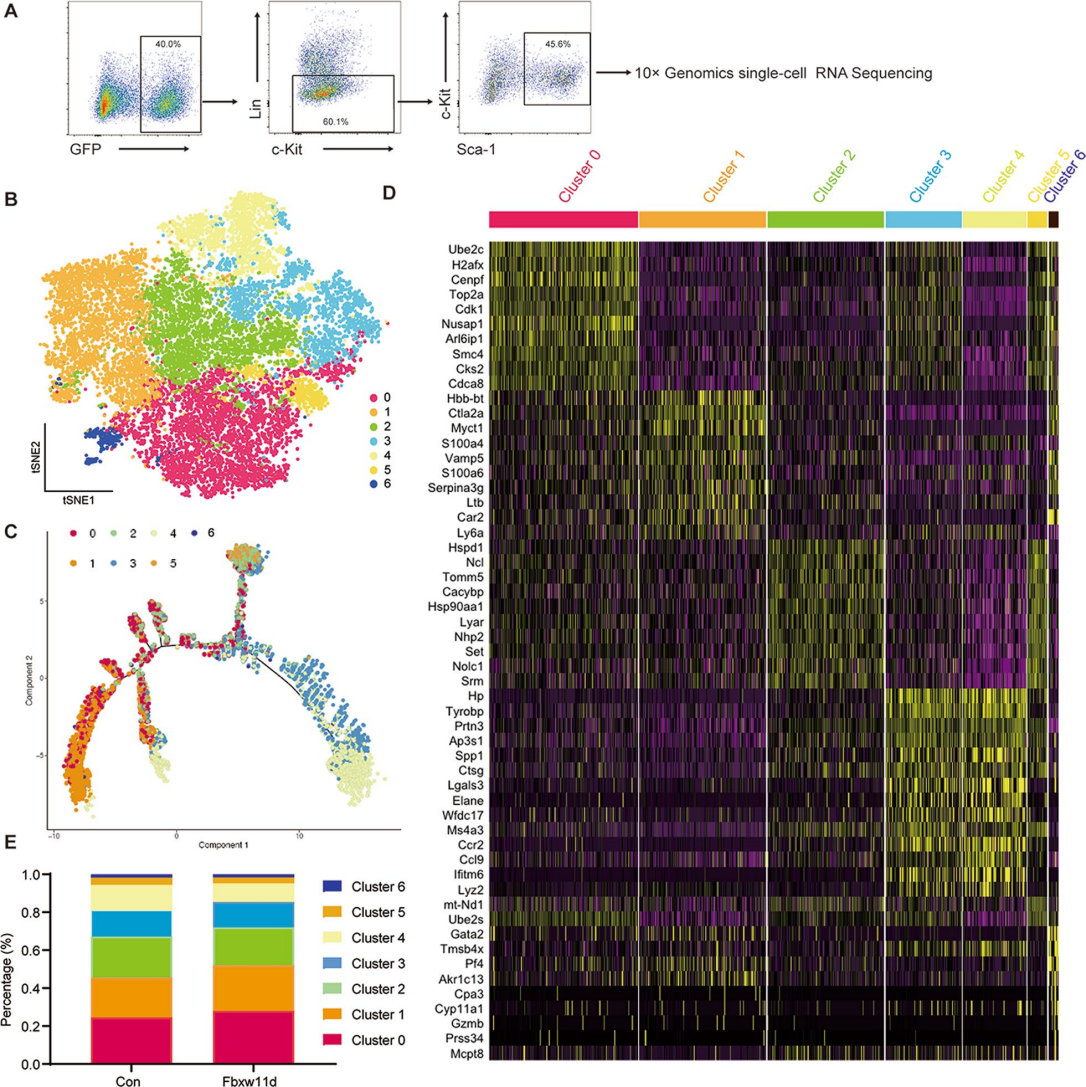
The effects of Fbxw11 on heterogeneous HSPC subpopulations at single-cell resolution. **A** Gating strategy for cell sorting. HSPCs were defined as live, GFP^+^Lin^–^c-Kit^+^Sca-1^+^ (LSK) cells. **B** In total, 12,229 successfully profiled LSK single cells (5297 LSK-Con and 6932 LSK-Fbxw11d) underwent *t*-SNE analysis. **C** Pseudotime analysis applied to the t-SNE/Seurat clusters defining their developmental relationship. **D** Heatmap shows scaled expression (log TPM values) of cluster-specific gene sets of each cluster in all clusters. AUC cutoff ≥ 0.85. For cluster 0 to cluster 3, only the top 10 cluster-specific genes are shown. **E** The distribution of each cluster in LSK-Con and LSK-Fbxw11d is shown

To determine the effects of Fbxw11 on the gene expression profile and composition of normal HSPCs, scRNA-seq was performed using 10× Genomics. As shown in Fig. 5A, LSK-Con and LSK-Fbxw11d were sorted after infection. In total, 5297 LSK-Con cells and 6932 LSK-Fbxw11d cells passed all the quality control filters and were first analyzed using Cell Ranger. These cells (12,229 cells) were put into one group for analyses. The median number of genes per cell was 4169. Principal component analysis (PCA) and t-SNE revealed significant transcriptional heterogeneity within LSK cells, which were divided into 7 major cell clusters (Fig. 5B). Trajectory analysis showed that cluster 1 and cluster 0 represented populations of more primitive stem cells, and other clusters were split into two branches which showed the myeloid and lymphoid lineages (Fig. 5C). The heatmap of cluster-specific genes (top 10 DEGs for cluster 0 to cluster 3) in different clusters is shown in Fig. 5D. Their expression is also shown as a violin plot (Additional file 1: Fig. S3).

To unravel which established HSPC populations corresponded to these cell clusters, two published scRNA-seq datasets were used as references [21, 22]. Population-specific genes of different transcriptionally defined HSPC populations, *i.e.,* tHSC1 to tHSC3 and tMPP1 to tMPP5, were obtained [21, 22], and the expression of the population-specific genes in 7 cell clusters is shown in heatmaps (Additional file 1: Fig. S4). Cluster 1 corresponded to primitive HSCs since they expressed higher levels of tHSC1- and tHSC2-specific genes. Furthermore, among the top ten specific genes of cell cluster 1, *Myct1* and *Ly6a* were tHSC1-specific genes, and *Ltb* was a tHSC2-specific gene [22]. In addition, expression of the top ten specific genes of cell cluster 1 was also analyzed by the published Web tool (http://blood.stemcells.cam.ac.uk/single_cell_atlas.html) [21]. Three genes, *i.e., Ltb*, *Myct1* and *Serpina3g*, were mainly expressed in more primitive HSCs, including LT-HSCs (Additional file 1: Fig. S5). It is worth noting that cluster 1 cells expressed high levels of *Hbb-bt*, *Ctla2a*, *S100a4*, *Vamp5*, *S100a6* and *Car2*, which did not appear in the population-specific genes tHSC1, tHSC1 [21, 22] or LT-HSC [21]. Cluster 0 corresponded to the tMPP1 population [22]. They shared specific genes, *i.e., Ube2c*, *Cenpf*, *Top2a*, *Cdk1* and *Nusap1*. Cluster 0 also had unique specific genes, such as *H2afx*, *Arl6ip1*, *Smc4*, *Cks2* and *Cdca8*. Cluster 2 did not seem to correspond to any tHSCs or tMPPs, although they shared specific genes, *i.e., Nolc1* and *Srm*, with tMPP5 [22]. Cluster 2 also had unique specific genes, including *Hspd1*, *Ncl*, *Tomm5*, *Cacybp*, *Hsp90aa1*, *Lyar*, *Nhp2* and *Set*. Although cluster 3 and cluster 4 corresponded to tHSC3, they had significant enrichment of myeloid progenitor gene sets, including *Hp*, *Prtn3*, *Ctsg*, *Elane*, *Ms4a3* and *Ccr2*. Cluster 6 expressed a high level of *Pf4* and exhibited positive enrichment of the megakaryocyte progenitor cell gene sets (Fig. 5D).

The percentage of each cluster was calculated and plotted to determine whether overexpression of Fbxw11 affected the distribution of different clusters. No significant difference was detected between the LSK-Con and LSK-Fbxw11d groups (Fig. 5E). These data indicated that Fbxw11 had little effect on the composition of heterogeneous HSPC subpopulations, although it dramatically decreased the absolute number of HSPCs.

### Single-cell analysis reveals the effects of Fbxw11 on the expression of genes associated with HSC maintenance and lineage differentiation

**Fig. 6:**
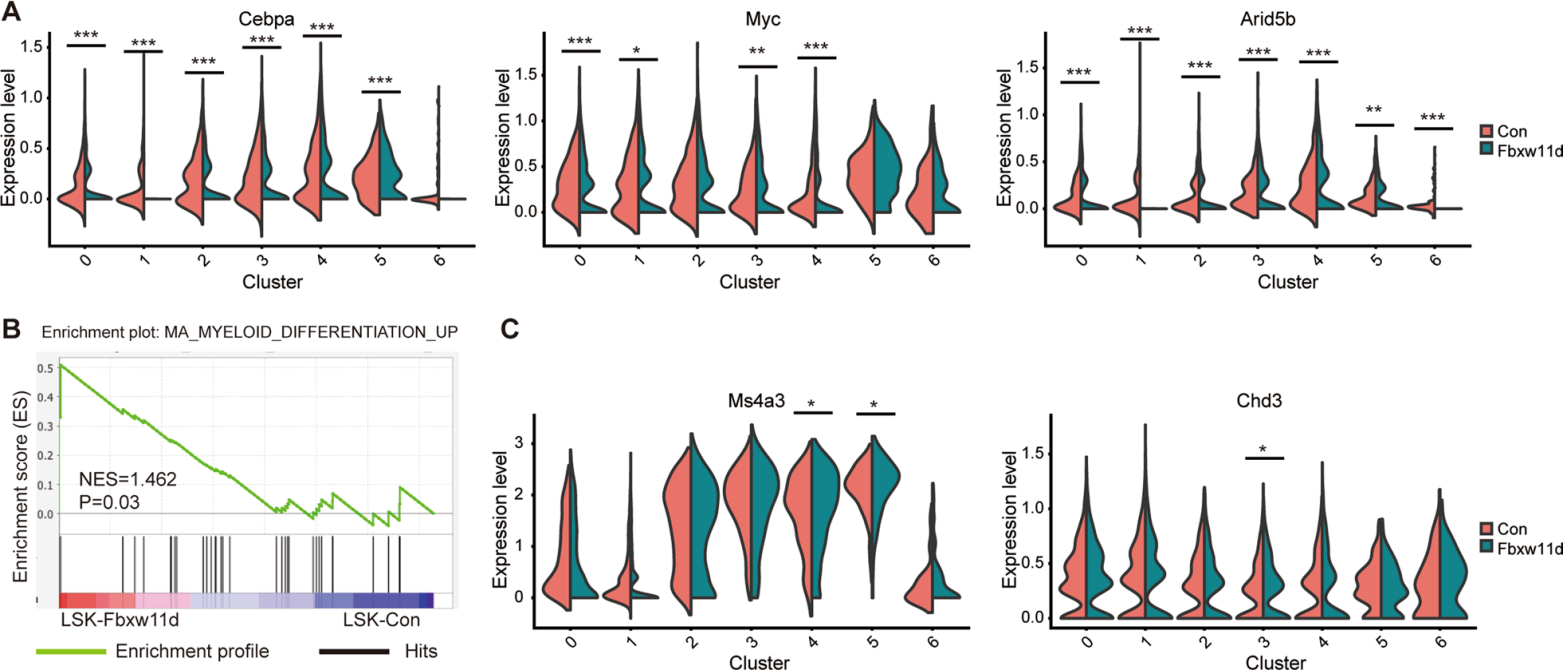
The effects of Fbxw11 on the expression of genes associated with HSC maintenance and granulocytic lineage-biased differentiation. **A** Violin plots demonstrate the expression of Cebpa, Myc, Arid5b and Apoe in clusters in LSK-Con and LSK-Fbx11d. **B** DEGs underwent GSEA analysis, and the reported signature of “myeloid differentiation up” is shown. **C** Violin plots demonstrate the expression of Ms4a3 and Chd3 in clusters from LSK-Con and LSK-Fbxw11d. *p < 0.05, **p < 0.01, ***p < 0.001

To further explore which genes are affected by Fbxw11 overexpression at single-cell level, we focused on genes that were differentially expressed between LSK-Fbxw11d and LSK-Con, especially transcription factors (TFs). *Cebpa*, *Myc* and *Arid5b*, which significantly regulate HSPC activity, were downregulated in almost all clusters of LSK-Fbxw11d when compared with LSK-Con (Fig. 6A). Gene set enrichment analysis (GSEA) showed enrichment of “myeloid differentiation up” in LSK-Fbxw11d compared with LSK-Con (Fig. 6B). Myeloid development-associated TFs and specific genes were also analyzed based on scRNA-seq results. *Ms4a3* and *Chd3*, which are important for myeloid development [36], were highly expressed in myeloid prone subpopulations of LSK-Fbxw11d (Fig. 6C). Myeloid-associated TFs *Stat3* and *Etv6* were also slightly upregulated, though the percentage of positive cells was lower (Additional file 1: Fig. S6). Collectively, these results suggested that Fbxw11 might exert its regulatory effects on HSC maintenance and lineage differentiation through the regulation of self-renewal and myeloid-associated TFs.

## Discussion

Stem cells are required for tissue maintenance. Their self-renewal potential and pluripotency are tightly regulated by sophisticated signal transduction networks. Accumulating evidence has revealed that the self-renewal, pluripotency and differentiation of stem cells are precisely controlled by the UPS system, which controls highly ordered protein degradation [37, 38]. Abnormal proteasome activity can lead to serious consequences. The E3 ligases in the UPS act as recognition components, and different members have heterogeneous effects on stem cells by targeting diverse spectra of proteins. While abnormal control of ubiquitination contributes to the initiation and progression of malignancies, mounting evidence suggests that dysfunction of the UPS is also related to the failure of stem cell self-renewal and functions. Abnormal ubiquitination in embryonic stem cells impairs their potential to differentiate into neural cells and causes fetal death [37, 39]. Abnormal control of ubiquitination in the hematopoietic system is related to Fanconi anemia and aplastic anemia [40–42]. In the present study, we show that increased expression of Fbxw11 results in impaired stem cell maintenance. Our work provides a deeper understanding of the UPS-mediated regulatory mechanism in hematopoiesis.

Quiescence and differentiation are two important factors to maintain HSCs homeostasis. Quiescence is necessary for maintaining the stemness and function of HSCs under steady-state conditions. Breaking quiescence leads to HSC exhaustion and eventually causes failure of hematopoiesis [43]. Our observations suggested that Fbxw11 decreased the quiescence of HSCs by pushing them into the cell cycle, which impaired the stemness and sustained the self-maintenance potential of HSCs. Differentiation-associated mechanisms may also be involved in this process since we got the following observations. First, GMPs expressed the highest level of Fbxw11 among HSPC subpopulations. Second, Fbxw11 increased the proportion of GMPs and CFU-GM colony in vitro, while it increased the percentage of Gr-1^+^CD11b^+^ granulocytic cells and decreased the percentage of CD3^+^ or B220^+^ lymphocytes in PB, BM and SP after transplantation in vivo. Third, Fbxw11-overexpressing LSK cells expressed higher levels of Csf1r, Csf2r, Runx1 and Stat3, which are important for myeloid development [44–47]. Fourth, GSEA results also demonstrated the enrichment of “myeloid differentiation up” associate gene. It is possible that Fbxw11 might promote myeloid differentiation of HSCs, which may also account for the impaired repopulation capacity of HSCs. Unfortunately, we do not have direct evidence, such as the GMP output after transplantation in vivo, due to the very low graft reconstruction status.

Although the UPS affects the repopulation and function of stem cells through different mechanisms, reshaping the expression spectrum of TFs is one of the most important steps. In fact, some TFs that are important for stem cells are direct targets of the UPS. The UPS maintains the balance of self-renewal and differentiation of stem cells by maintaining the stability of key TFs, such as Myc and OCT-3/4, which are directly recognized by Fbxw7 and WWP2 E3 ligases [48, 49]. However, in many cases, there is no direct link between abnormal E3 ligase and altered TFs. In these cases, UPS indirectly affects TFs through broad-spectrum and sophisticated signal transduction pathways [39, 50]. Here, we found that the expression of *Cebpa*, *Myc* and *Arid5b* in HSPCs was sharply decreased by elevated expression of Fbxw11. C/EBPα is an important transcriptional regulator in HSCs and plays roles not only in myeloid differentiation [51], but also in HSC quiescence. C/EBPα deletion was associated with a dramatic loss in HSC self-renewal. Furthermore, Cebpa^−/−^ HSCs failed to reconstruction in secondary recipients or in irradiated mice in non-competitive transplantation experiments. Therefore, C/EBPα is indeed essential for HSC function, at least during hematopoietic reconstitution [52, 53]. Myc proteins (c-Myc and N-Myc) are downstream targets of C/EBPα and are necessary for the proliferation and survival of HSCs [54, 55]. Arid5b is a critical downstream target of TAL1 and is essential for maintaining the multipotency and quiescence of HSCs [56, 57]. Hence, Fbxw11 may cause failure of HSPCs through the downregulation of these TFs.

HSPCs are composed of hierarchically heterogeneous subpopulations [58]. Whether Fbxw11 specifically modulates some subpopulations or has similar effects on most subpopulations has not been established. Compared with conventional bulk RNA-seq, which captures the average gene expression profile of all subpopulations, the scRNA-seq technique is capable of resolving heterogeneity among subpopulations and acquiring gene profiling at the single-cell level. Hence, scRNA-seq was carried out to reveal the heterogeneity within HSPCs as well as to show the effects of Fbxw11 on the gene expression profiles of different subpopulations. With the development of scRNA-seq techniques, several groups have revealed transcriptional profiles of HSPC subpopulations following the strategy that each predetermined subpopulation was first obtained by accurate sorting based on established surface markers before scRNA-seq [21, 22, 59]. Here, we followed another strategy in which all LSK cells underwent scRNA-seq, and cell clusters were entirely determined by gene expression profiles. We believe that this strategy more accurately reflects the nature of LSK cells since it avoids missing transitional cells and eliminates possible defects by arbitrary predetermination of HSPC subpopulations. We classified seven major cell clusters by transcriptome landscaping. Although these clusters share some identical specific genes with HSPC subpopulations reported in the literature, they also have unique specific genes. Hence, our findings contribute to a better understanding of HSPC heterogeneity and provide reference for further definition of HSPC subpopulations. Functional assays in the future will further classify these clusters. Nevertheless, an important result we obtained is that Fbxw11 has little effect on the composition of heterogeneous HSPC subpopulations, although it dramatically decreases the absolute number of HSPCs, suggesting that Fbxw11 may have similar effects on all HSPC subpopulations. The observation that C/EBPα, Myc and Arid5b, self-renewal-associated TFs, were downregulated in all HSPC clusters overexpressing Fbxw11 further confirmed this conclusion. The above results suggest that elevated Fbxw11-related UPS decreases the self-renewal potential of all HSPC subpopulations, which results in the failure of HSCs and low graft reconstruction.

## Conclusion

In summary, our work elucidates the novel role of Fbxw11 in the regulation of HSPCs and reveals the transcriptomic heterogeneity of HSPCs at the single-cell level. High-level expression of Fbxw11 decreased the expression of key molecules in the regulation of self-renewal of HSPCs, *i.e.,* C/EBPα, Myc and Arid5b, in all HSPC subpopulations; reduced quiescence and reconstruction of HSPCs; and finally caused failure of HSCs. These results provide additional views on the role of ubiquitin in hematopoietic stem cell maintenance.

## Supplementary Information

**Additional file 1: Fig. S1.** The strategies to analyze and sort hematopoietic cell populations used in this study by FACS are shown. (**A**) LK cells, LSK cells, LT-HSCs, ST-HSCs, MPPs, CMPs, GMPs, MEPs were sorted after enrichment using c-Kit^+^ magnetic beads. (**B**) Common lymphoid progenitors (CLPs) were also sorted after enrichment using c-Kit^+^ magnetic beads. (**C**) Gating strategy of granulocytes. (**D**) Gating strategy of monocytes and macrophages. (**E**) Gating strategy of B cells and T cells. (**F**) Gating strategy of NK cells. **Fig. S2.** (**A**) Lethally irradiated C57BL/6 mice were transplanted with 1 × 10^6^ GFP^+^ LK cells (Lin^-^ c-Kit^+^) from the control and Fbxw11 groups. Cells homing to the BM were analyzed by FACS 16 hours after transplantation (control: n = 3; Fbxw11d: n = 7). (**B**–**D**) The overall reconstitution status (including both GFP^+^ cells and competitor cells) was assessed at the 1st, 3rd, and 5th month. (**E**) The percentage of donor LSK cells in BM at the 5th month after first-round transplantation was analyzed by FACS, n = 3. **Fig. S3.** Violin plots show the expression of cluster-specific genes of each cluster in all clusters. For cluster 0 to cluster 3, only the top 10 cluster-specific genes are shown. **Fig. S4.** The heatmaps show the expression of population-specific genes of transcriptionally defined HSPC populations from published references in LSK-Con and LSK-Fbxw11d (Dong’s paper, PMID: 32367048). **Fig. S5.** Expression of cluster 1-specific genes (Ltb, Myct1 and Serpina3g) in LT-HSCs in the published reference model (Nestorowa’s paper, PMID: 27365425). **Fig. S6.** Violin plots demonstrate the expression of Stat3, Etv6 and Chd3 in clusters from LSK-C and LSK-F11D. *p < 0.05, **p < 0.01, ***p < 0.001.

**Additional file 2. Table S1.** Antibldies list.

**Additional file 3. Table S2.** Primers used for detection of mouse genes by real-time RT-PCR analysis.

## Data Availability

The datasets used and/or analyzed during the current study are available from the corresponding author on reasonable request.

## Abbreviations

HSPCs: Hematopoietic stem/progenitor cells
scRNA-seq: Single-cell RNA sequencing
LSK: Lin^−^c-Kit^+^Sca-1^+^
LK: Lin-c^−^kit^+^
HSCs: Hematopoietic stem cells
UPS: Ubiquitin–proteasome system
LT-HSCs: Long-term hematopoietic stem cells
ST-HSCs: Short-term hematopoietic stem cells
MPPs: Multipotent progenitors
CMPs: Common myeloid progenitors
GMPs: Granulocyte–monocyte progenitors
MEPs: Megakaryocyte–erythroid progenitors
CLPs: Common lymphoid progenitors
GEO: Gene Expression Omnibus
DEGs: Differentially expressed genes
TFs: Transcription factors
GSEA: Gene set enrichment analysis
