# Supplementary figures and images for: Fbxw11 impairs the repopulation capacity of hematopoietic stem/progenitor cells
Source: Stem Cell Res Ther. 2022 Jun 11;13:245. doi: 10.1186/s13287-022-02926-9 (PMC9188144; doi:10.1186/s13287-022-02926-9)

**A**

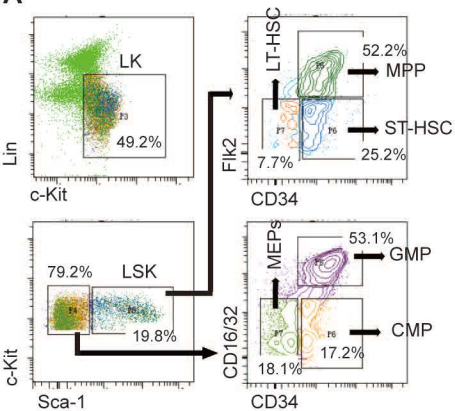

**B**

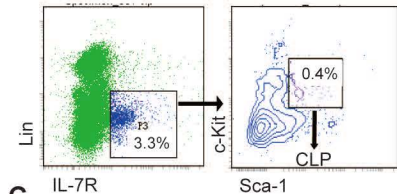

**C**

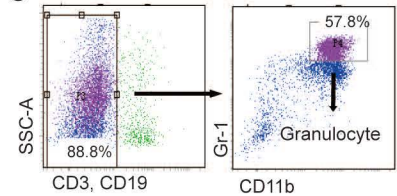

**D**

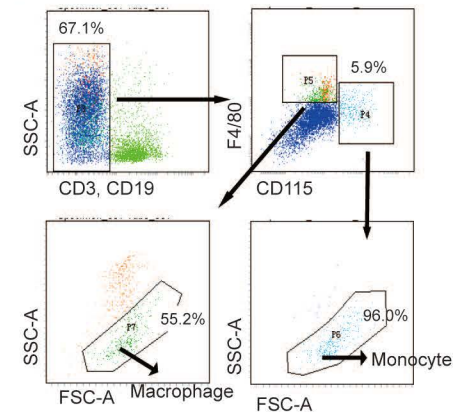

**E**

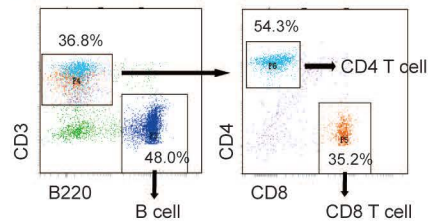

**F**

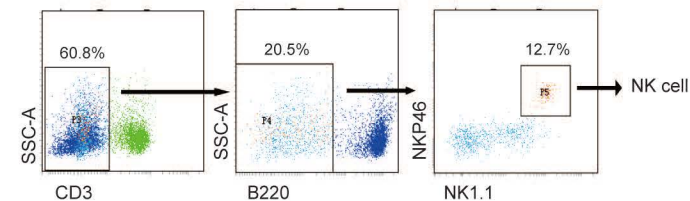

**A**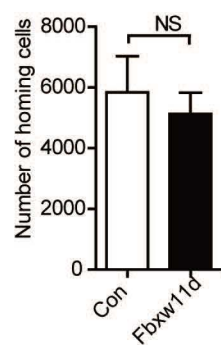**B**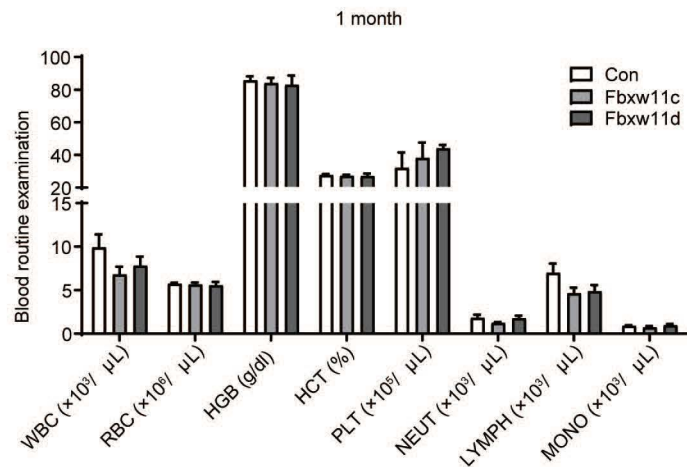**C**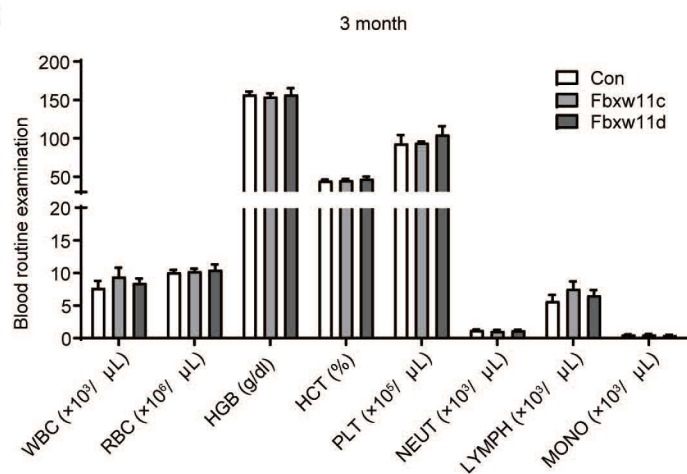**D**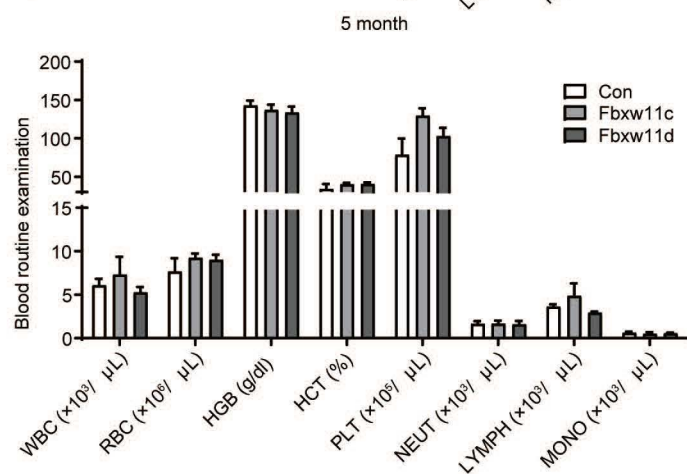**E**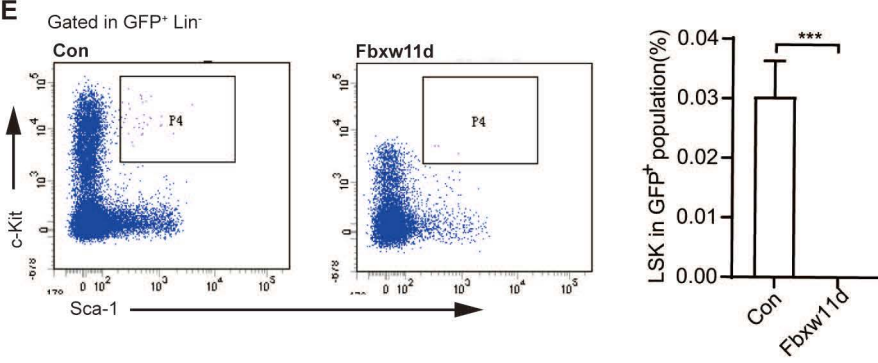

A

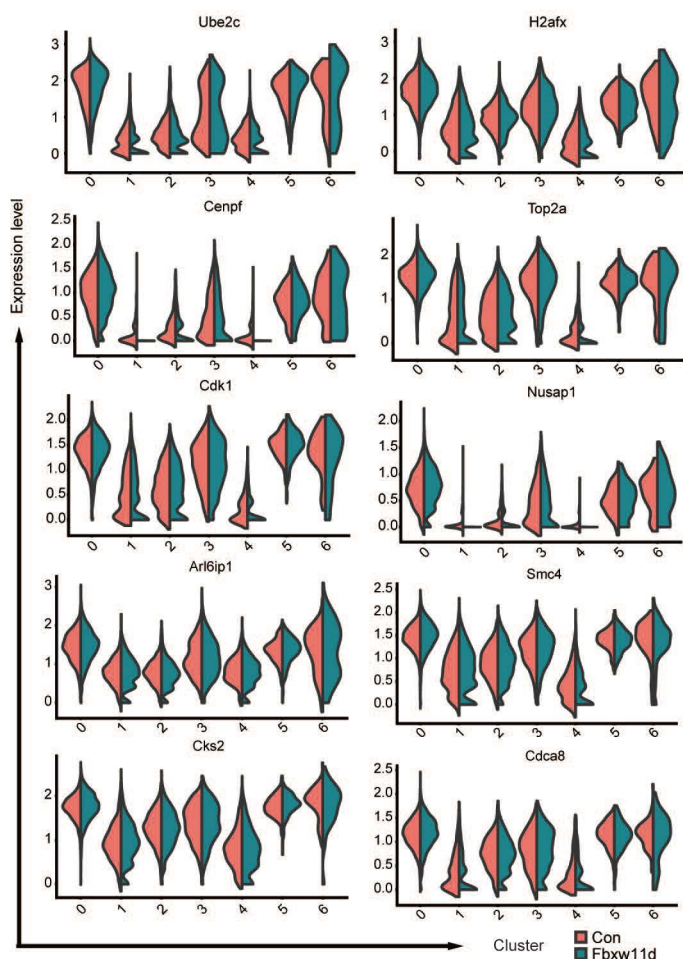

B

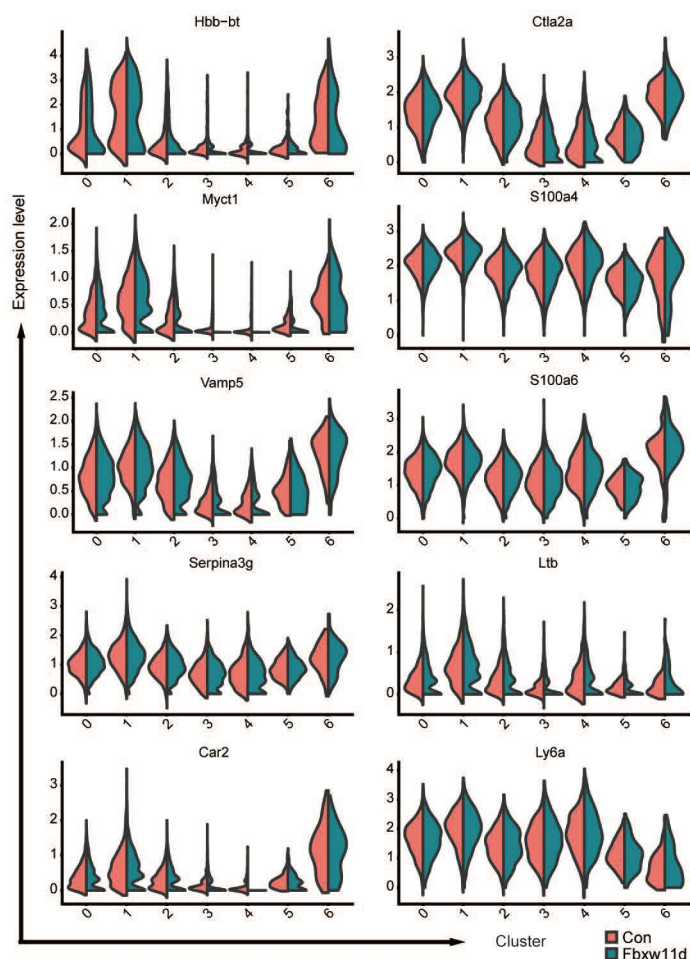

C

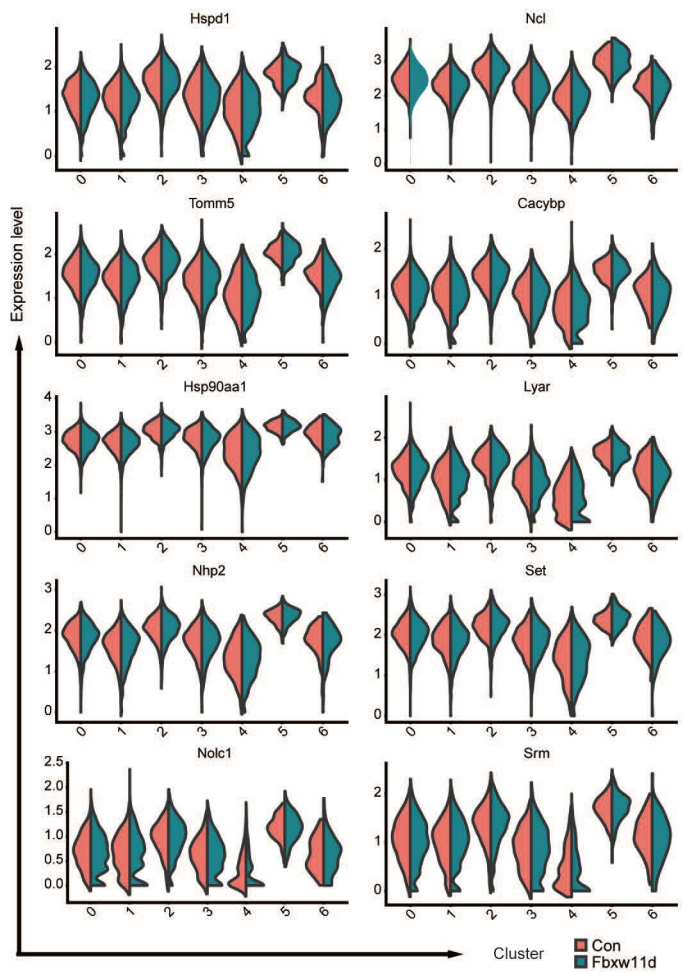

D

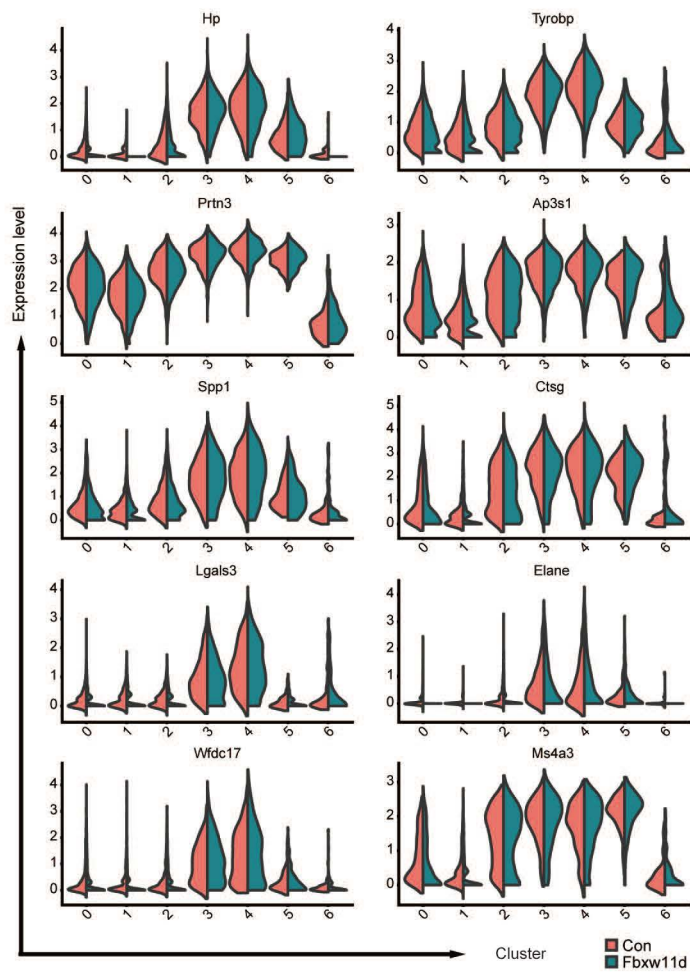

E

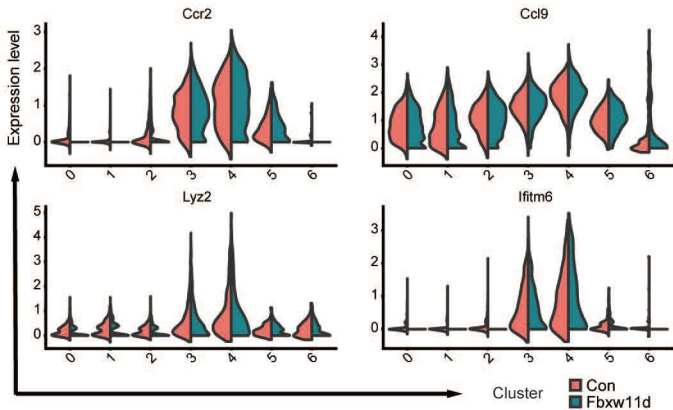

F

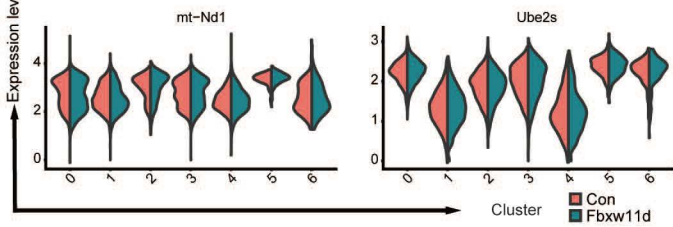

G

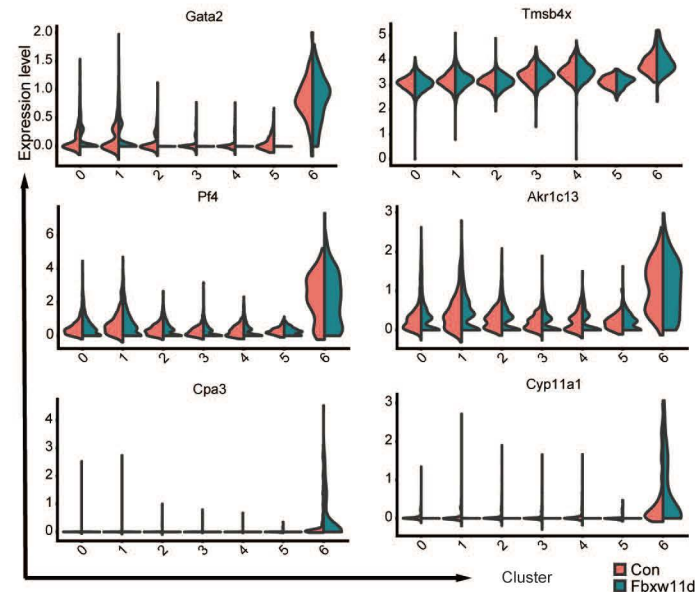

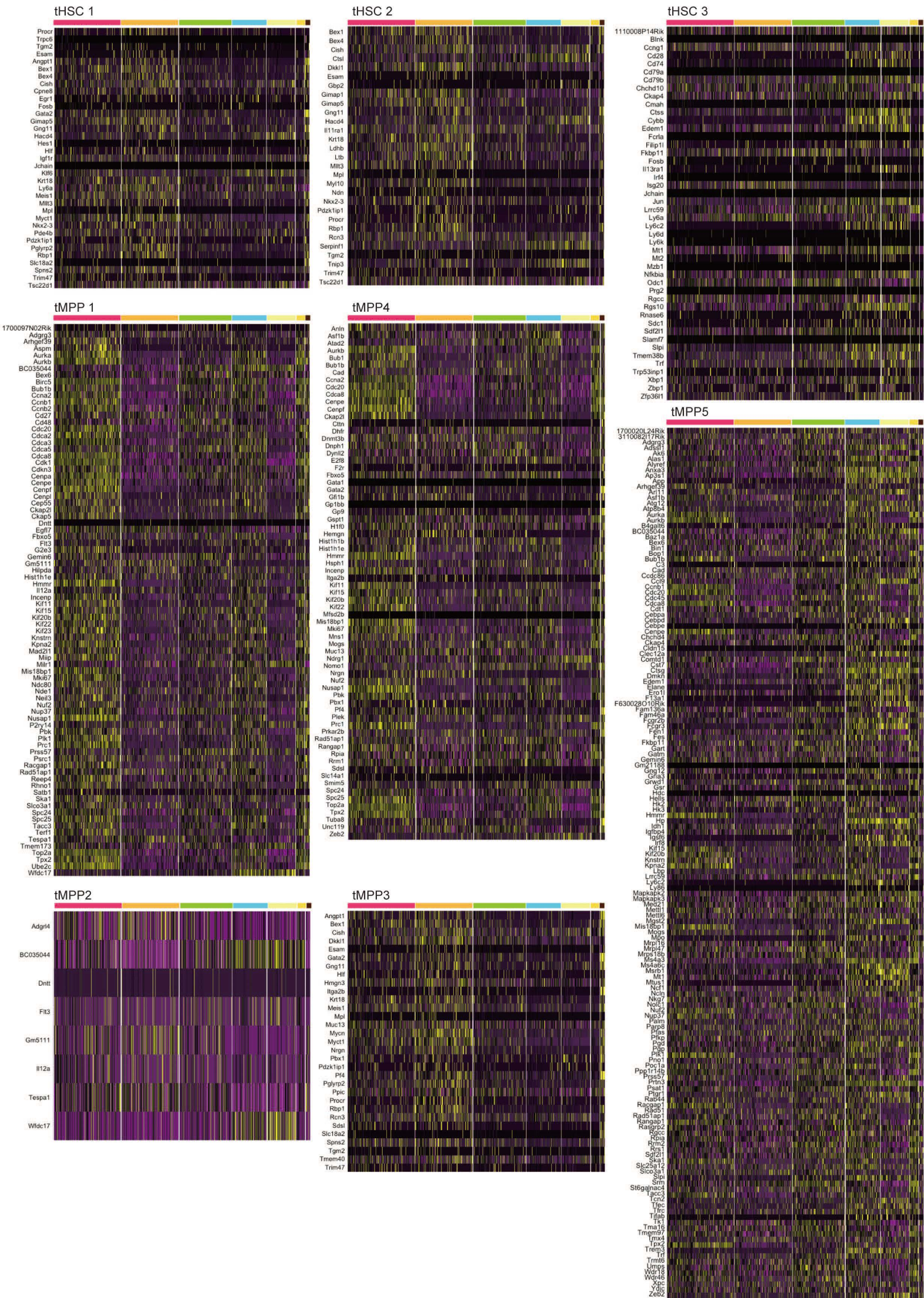

**A**

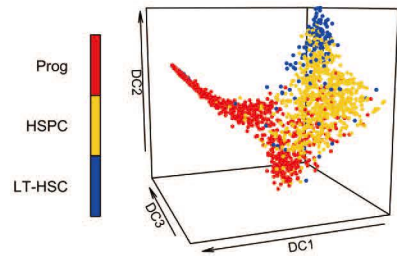

**B**

LTB - ENSMUSG00000024399

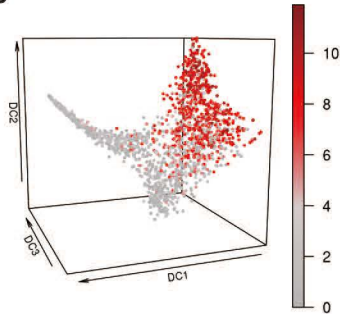

MYCT1 - ENSMUSG00000046916

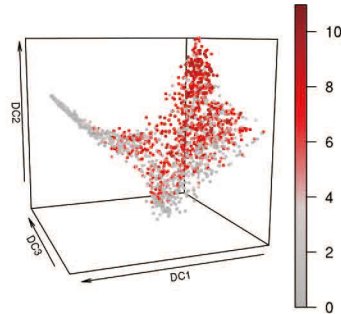

SERPINA3G - ENSMUSG00000041481

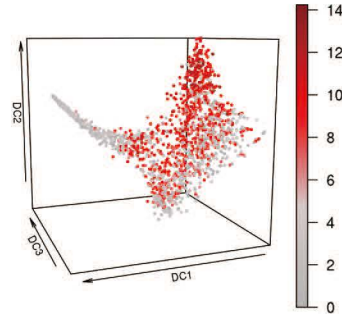

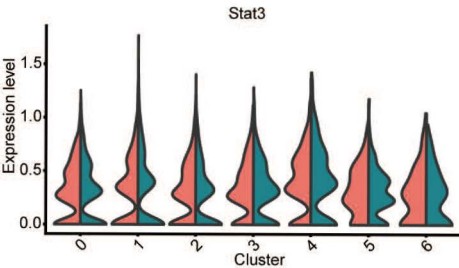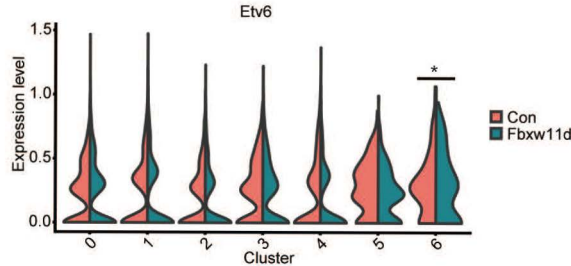

Supplement: Supplementary file 1 — Additional file 1: Fig. S1. The strategies to analyze and sort hematopoietic cell populations used in this study by FACS are shown. (A) LK cells, LSK cells, LT-HSCs, ST-HSCs, MPPs, CMPs, GMPs, MEPs were sorted after enrichment using c-Kit+ magnetic beads. (B) Common lymphoid progenitors (CLPs) were also sorted after enrichment using c-Kit+ magnetic beads. (C) Gating strategy of granulocytes. (D) Gating strategy of monocytes and macrophages. (E) Gating strategy of B cells and T cells. (F) Gating strategy of NK cells. Fig. S2. (A) Lethally irradiated C57BL/6 mice were transplanted with 1 × 10^6^ GFP+ LK cells (Lin- c-Kit+) from the control and Fbxw11 groups. Cells homing to the BM were analyzed by FACS 16 hours after transplantation (control: n = 3; Fbxw11d: n = 7). (B–D) The overall reconstitution status (including both GFP+ cells and competitor cells) was assessed at the 1st, 3rd, and 5th month. (E) The percentage of donor LSK cells in BM at the 5th month after first-round transplantation was analyzed by FACS, n = 3. Fig. S3. Violin plots show the expression of cluster-specific genes of each cluster in all clusters. For cluster 0 to cluster 3, only the top 10 cluster-specific genes are shown. Fig. S4. The heatmaps show the expression of population-specific genes of transcriptionally defined HSPC populations from published references in LSK-Con and LSK-Fbxw11d (Dong’s paper, PMID: 32367048). Fig. S5. Expression of cluster 1-specific genes (Ltb, Myct1 and Serpina3g) in LT-HSCs in the published reference model (Nestorowa’s paper, PMID: 27365425). Fig. S6. Violin plots demonstrate the expression of Stat3, Etv6 and Chd3 in clusters from LSK-C and LSK-F11D. *p < 0.05, **p < 0.01, ***p < 0.001. [file 13287_2022_2926_MOESM1_ESM.pdf]
