# Supplementary material for: Fbxw11 impairs the repopulation capacity of hematopoietic stem/progenitor cells
Source: Stem Cell Res Ther. 2022 Jun 11;13:245. doi: 10.1186/s13287-022-02926-9 (PMC9188144; doi:10.1186/s13287-022-02926-9)
Supplement: Supplementary file 2 — Additional file 2. Table S1. Antibldies list. [file 13287_2022_2926_MOESM2_ESM.docx]

**Table S1 Antibldies list**

| Marker | Fluorophore | Clone | Source |
| --- | --- | --- | --- |
| Lin | V450 |  | BD bioscience |
| c-kit (CD117) | PE-Cy7 | 2B8 | Biolegend |
| Sca-1 | APC | D7 | Biolegend |
| CD3 | PerCP-Cy5.5 | 145-2C11 | BD bioscience |
| CD19 | PerCP-Cy5.5 | 1D3 | BD bioscience |
| B220 | PE | RA3-6B2 | Biolegend |
| F4/80 | APC | BM8 | eBioscience |
| CD11b | PerCP-Cy5.5 | M1/70 | BD bioscience |
| Gr1 | PE-Cy7 | RB6-8C5 | Biolegend |
| CD115 | PE | AFS98 | Biolegend |
| CD4 | PE-Cy7 | GK1.5 | Biolegend |
| CD8a | APC | 53-6.7 | Biolegend |
| NK1.1 | PE-Cy7 | PK136 | BD bioscience |
| NKp46(CD335) | APC | 29A14 | Biolegend |
| CD34 | PerCP-Cy5.5 | HM34 | Biolegend |
| CD16/32 | PE | 93 | Biolegend |
| IL7Ra (CD127) | PE | A7R34 | Biolegend |
| Flk2 | PE | A2F10 | Biolegend |
| Ki-67 | PE | 16A8 | Biolegend |
