# Supplementary material for: Fbxw11 impairs the repopulation capacity of hematopoietic stem/progenitor cells
Source: Stem Cell Res Ther. 2022 Jun 11;13:245. doi: 10.1186/s13287-022-02926-9 (PMC9188144; doi:10.1186/s13287-022-02926-9)
Supplement: Supplementary file 3 — Additional file 3. Table S2. Primers used for detection of mouse genes by real-time RT-PCR analysis. [file 13287_2022_2926_MOESM3_ESM.docx]

**Table S2** Primers used for detection of mouse genes by real-time RT-PCR analysis

| Gene name | Forward primer | Reverse primer |
| --- | --- | --- |
| Fbxw11 | CGGGACTTTATCACTGCTTTA | ATCACTCGCTGCCATTCTTTA |
| GAPDH | CACTTGAAGGGTGGAGC | GGGCTAAGCAGTTGGTG |
| Cebpa | GCGGGCAAAGCCAAGAAGTCG | GGCGGTCATTGTCACTGGTCAACTC |
| Runx1 | CTGCTCCGTGCTACCCACTCAC | CTGGATCTGCCTGGCATTTCG |
| Csf1r | GTGGAGACACCAAGCTGGAA | CTGTCACCCACAGACACCTC |
| Csf2r | CCCTGCTCTTCTCCACGCTACTGC | CGACCGTGCCATTGACATCCAG |
| HoxA5 | CTCATTTTGCGGTCGCTATCC | ATCCATGCCATTGTAGCCGTA |
| Hoxb13 | GGCTACCTACCCTTCGGAAACT | TTCACCTTGGCAACACATCTGG |
| Myc | CCCTATTTCATCTGCGACGAG | GAGAAGGACGTAGCGACCG |
| Oct4 | GGAATCGGACCAGGCTCAGA | CCTTCTCCAACTTCACGGCATTG |
| Sox2 | CGCACATGAACGGCTGGAG | GCCTCGGACTTGACCACAGA |
| Tim1 | ACATATCGTGGAATCACAACGAC | ACTGCTCTTCTGATAGGTGACA |
| ClylinD3 | TCTTCCCTCTGGCTATGAACTAC | AGCACCTCCCACTCCCGCAACTG |
| Cdkn1c | GAGCCGTCCATCACCAAT | TGTCCACCTCCATCCACTG |
